# Supplementary material for: The cytokines HGF and CXCL13 predict the severity and the mortality in COVID-19 patients
Source: Nat Commun. 2021 Aug 9;12:4888. doi: 10.1038/s41467-021-25191-5 (PMC8352963; doi:10.1038/s41467-021-25191-5)
Supplement: Supplementary file 1 — Supplementary Information [file 41467_2021_25191_MOESM1_ESM.pdf]

Supplementary Table 1. Clinical and laboratory characteristics of included patients on admission, according to ICU stay

	**Non ICU**	**ICU**	**Total**	***P* Value**
n	(N=55)	(N=43)	(N=98)	
Male sex - no. (%)	34 (61.8)	31 (72.1)	65 (66.3)	0.285
Median age, years (IQR)	68 (55 - 80)	60 (56 - 69)	63.5 (56 - 74)	0.077
Body mass index, kg/m^2 (IQR)	27.0 (22.5-30.0)	27.8 (24.9-33.3)	27.1 (23.7-31.3)	0.116
Obesity - no. (%)	14 (26.4)	18 (41.9)	32 (33.3)	0.110
Comorbidities - no. (%)	48 (87.3)	34 (79.1)	82 (83.7)	0.276
Hypertension	28 (50.9)	25 (58.1)	53 (54.1)	0.476
Coronary artery disease	9 (16.4)	4 (9.3)	13 (13.3)	0.378 §
Chronic kidney disease	12 (21.8)	5 (11.6)	17 (17.4)	0.186
Chronic obstructive pulmonary disease	6 (10.9)	3 (7.0)	9 (9.2)	0.727 §
Asthma	3 (5.5)	4 (9.3)	7 (7.1)	0.696
Diabetes	18 (32.7)	11 (25.6)	29 (29.6)	0.442
Cirrhosis	2 (3.6)	0 (0.0)	2 (2.0)	0.502 §
Cancer	9 (16.4)	3 (7.0)	12 (12.2)	0.219 §
Median Charlson Comorbidity Index (IQR)	5 (2 - 7)	3 (2 - 5)	4 (2 - 6)	0.018
Ongoing therapy - no. (%)				
Immunosupressive drugs	5 (9.1)	1 (2.3)	6 (6.1)	0.226 §
RAAS inhibitors	12 (24.0)	19 (48.7)	31 (34.8)	0.015 §
Symptoms - no. (%)				
Fever	40 (72.7)	35 (81.4)	75 (76.5)	0.315
Fatigue	34 (61.8)	17 (39.5)	51 (52)	0.028
Anosmia/dysgueusia	7 (12.7)	3 (7.0)	10 (10.2)	0.505 §
Cough	43 (78.2)	34 (79.1)	77 (78.6)	0.915
Dyspnea	34 (61.8)	33 (76.7)	67 (68.4)	0.115
Arthralgia/myalgia	13 (23.6)	12 (27.9)	25 (25.5)	0.630
Nausea/vomiting	13 (23.6)	8 (18.6)	21 (21.4)	0.547
Vitals signs - median (IQR)				
Temperature (°C)	38.3 (37.6 - 38.7)	38.5 (37.9 - 38.9)	38.4 (37.7 - 38.9)	0.106
Systolic blood pressure (mm Hg)	115 (104 - 125)	109 (96 - 129)	113 (100 - 125)	0.169
Heart rate (bpm)	95 (86 - 106)	94.5 (85 - 113)	95 (86 - 107)	0.617
Respiratory rate (bpm)	25 (23 - 30)	31 (25 - 36)	28 (24 - 34)	0.002
qSOFA score ≥2	9 (16.4)	14 (35.9)	23 (24.5)	0.030
Laboratory findings - median (IQR)				
Oxygen saturation, percent	97 (95 - 99)	95 (93 - 98)	96 (95 - 99)	0.031
FiO_2, percent	21 (21 - 28)	43 (30 - 80)	29.5 (21 - 50)	1.5 E-06
White cell count, 10^9 per L	6.7 (4.7 - 8.8)	8.3 (6.3 - 10)	7.8 (5.2 - 9.2)	0.018
Lymphocytes count, 10^9 per L	0.9 (0.4 - 1.3)	0.9 (0.7 - 1.1)	0.9 (0.6 - 1.1)	0.303
Platelets count, 10^9 per L	208 (166 - 261)	204 (158 - 280)	205.5 (163 - 271)	0.702
D-dimer, ng/mL	983 (649 - 1680)	1146 (943 - 1989)	1123 (703 - 1858)	0.133

Creatinine, µmol/L	96 (77 - 126)	97 (82 - 125)	96.5 (80 - 125)	0.841
C-reactive protein, mg/L	74 (34 - 119)	155 (85 - 295)	99.5 (49 - 213)	2.6 E-04
Procalcitonin, µg/L	0.2 (0.1 - 0.4)	0.4 (0.2 - 0.7)	0.2 (0.1 - 0.5)	0.003
Ferritin, µg/L	913.5 (372 - 1516)	1643.5 (1292 - 3045)	1294 (633.5 - 1953.5)	1.5 E-05

BMI, body mass index; COPD, Chronic obstructive pulmonary disease; NSAID, non-steroid anti-inflammatory drugs; Renin–Angiotensin–Aldosterone System inhibitors, RAAS inhibitors. The oxygen saturation values correspond to SpO2 during the highest oxygen provision. Between-group comparisons performed using two-sided chi-square or two-sided Fisher’s exact test (§) for categorical variables and two-sided Kruskal-Wallis test for continuous variables.

Supplementary Table 2. Complications during follow-up

	**Non ICU**	**ICU**	**Total**	***P* Value**
n	55	43	98	
**Complications – no. (%)**				
Asthma or COPD exacerbation	1 (1.8)	2 (4.7)	3 (3.1)	0.580 §
Community acquired pneumonia	6 (10.9)	11 (25.6)	17 (17.4)	0.057
Hospital acquired pneumonia	7 (12.7)	29 (67.4)	36 (36.7)	<0.001
Acute respiratory distress syndrome	3 (5.5)	40 (93.0)	43 (43.9)	<0.001 §
Pneumothorax	0 (0)	3 (7.0)	3 (3.1)	0.081 §
Pulmonary embolism	4 (7.3)	10 (23.3)	14 (14.3)	0.040 §
Other thromboembolic event	2 (3.6)	6 (14.0)	8 (8.2)	0.133 §
Stroke	2 (3.6)	2 (4.7)	4 (4.1)	1.000 §
Rhythm disorder	8 (14.6)	11 (25.6)	19 (19.4)	0.170
Acute kidney injury	6 (10.9)	14 (32.6)	20 (20.4)	0.008
Septic shock	1 (1.8)	10 (23.3)	11 (11.2)	0.001 §
Acute hepatic injury	1 (1.8)	13 (30.2)	14 (14.3)	<0.001 §

Between-group comparisons performed using two-sided chi-square or two-sided Fisher's exact test (§)

Supplementary Table 3. Immunomodulating and antibiotics treatments received during follow-up

	**Non ICU**	**ICU**	**Total**	***P* Value**
n	55	43	98	
Tocilizumab - no. (%)	1 (1.8)	36 (83.7)	37 (37.8)	1.31×10^{-18}
Any antibiotic treatment - no. (%)	19 (34.6)	36 (83.7)	55 (56.1)	1.12×10^{-6}§

Between-group comparisons performed using two-sided chi-square or two-sided Fisher's exact test (§)

Supplementary Table 4. Clinical and laboratory characteristics of included patients on admission, according to ICU stay, France

	**Non ICU**	**ICU**	**Total**	***P* Value**
n	31	30	61	
Male sex - no. (%)	19 (61.3)	23 (76.7)	42 (68.9)	0.195
Median age, years (IQR)	57 (48-64)	71.5 (60-79)	61.5 (55-74)	<0.001
Obesity - no. (%)	4 (13.3)	7 (25.0)	11 (19.0)	0.325 §
Comorbidities - no. (%)				
Hypertension	8 (26.7)	13 (46.4)	21 (36.2)	0.118
Asthma	3 (10.0)	2 (7.1)	5 (8.6)	1.000 §
Diabetes	6 (20.0)	5 (17.9)	11 (19)	0.835
Ongoing therapy - no. (%)				
Antiviral agents	18 (64.3)	9 (39.1)	27 (52.9)	0.073
Of which				0.407 §
Lopinavir/ritonavir	16 (88.9)	8 (88.9)	24 (88.9)	
Other	2 (11.1)	0 (0)	2 (7.4)	
Remdesivir	0 (0)	1 (11.1)	1 (3.7)	
Antibiotics	20 (71.4)	14 (60.9)	34 (66.7)	0.426
Symptoms - no. (%)				
Fever	28 (93.3)	18 (72.0)	46 (83.6)	0.064 §
Fatigue	24 (80.0)	16 (64.0)	40 (72.7)	0.185
Cough	24 (80.0)	15 (60.0)	39 (70.9)	0.104
Dyspnea	12 (40.0)	14 (56.0)	26 (47.3)	0.237
Arthralgia/myalgia	15 (50.0)	10 (40.0)	25 (45.5)	0.458
Nausea/vomiting	4 (13.3)	1 (4.0)	5 (9.1)	0.362 §
Vitals signs - median (IQR)				
Systolic blood pressure (mm Hg)	134 (117-138)	136 (118-146.5)	134 (117-146)	0.590
Heart rate (bpm)	91 (78-100.5)	85 (69-99)	90 (74-99)	0.333
Respiratory rate (bpm)	20 (16-24)	26 (22-34)	24.5 (20-34)	0.053
Laboratory findings - median (IQR)				
Oxygen saturation, percent	95 (93.5-97)	94 (90-96)	95 (92-96)	0.065
White cell count, 10^9 per L	4.5 (3.7-6.1)	6.4 (5.0-12.4)	5.6 (4.9-7.8)	0.009
Platelets count, 10^9 per L	136.5 (136-213)	181 (152-297)	173 (141-244)	0.055
C-reactive protein, mg/L	21 (7-76.9)	137 (66-174)	94.8 (27.3-172.6)	0.020

Between-group comparisons performed using two-sided chi-square or two-sided Fisher's exact test (§) for categorical variables and two-sided Kruskal-Wallis test for continuous variables.

Supplementary Table 5

Antibodies. The following antibodies were used for mass cytometry experiments. Panel 1: 111Cd-conjugated anti-CD141 (1A4), 113In-conjugated anti-CD8 (RPA-T8), 115In-conjugated anti-CD4 (RPA-T4), 116Cd-conjugated anti-IgA2 (A9604D2), 141Pr-conjugated anti-CD45 (HI30), 142Nd-conjugated anti-CD19 (HIB19), 143Nd-conjugated anti-ICOS (C398.4A), 144Nd-conjugated anti-IgG3 (HP6047), 145Nd-conjugated anti-CD31/PECAM-1 (WM59), 146Nd-conjugated anti-IgD (IA6-2), 147Sm-conjugated anti-CD7 (CD7-6B7), 148Nd-conjugated anti-IgA1 (B3506B4), 149Sm-conjugated anti-CD127 (A019D5), 150Nd-conjugated anti-IgG1 (G17-1), 151Eu-conjugated anti-CD123 (6H6), 152Sm-conjugated anti-CD21 (BL13), 153Eu-conjugated anti-CD62L (DREG-56), 154Sm-conjugated anti-CD3 (UCHT1), 155Gd-conjugated anti-CD27 (L128), 156Gd-conjugated anti-TCR γ (gamma)/δ (delta) (B1), 158Gd-conjugated anti-CD10 (HI10a), 159Tb-conjugated anti-CD197/CCR7 (G043H7), 160Gd-conjugated anti-CD14 (M5E2), 161Dy-conjugated anti-CD1c (L161), 162Dy-conjugated anti-CD11c (Bu15), 163Dy-conjugated anti-CD183/CXCR3 (G025H7), 164Dy-conjugated anti-CD185/CXCR5 (51505), 165Ho-conjugated anti-CD45RO (UCHL1), 166Er-conjugated anti-CD24 (ML5), 167Er -conjugated anti-CD38 (HIT2), 168Er-conjugated anti-CD66b (G10F5), 169Tm-conjugated anti-CD25 (2A3), 170Er-conjugated anti-CD45RA (HI100), 171Yb-conjugated anti-CD20 (2H7), 172Yb-conjugated anti-IgM (MHM-88), 173Yb-conjugated anti-TCR α(alpha)/ β(beta) (T1089.A-31), 174Yb-conjugated anti-HLA-DR (L243), 175Lu-conjugated anti-CD279/PD-1 (EH12.2H7), 176Yb-conjugated anti-CD56 (HCD56), 198Pt-conjugated anti-IgG2 (HP6002), 209Bi-conjugated anti-CD16 (3G8), 112Cd-conjugated anti-CD69 (FN50), 106Cd-conjugated anti-CCR6 (11A9), 194Pt-conjugated anti-CCR4 (L291H4) and 191Ir was used to label DNA. Antibodies against TCR α(alpha)/ β(beta), IgG1, CD66b, CCR6 and CD141 were purchased from BD. Antibodies against TCR γ (gamma)/δ (delta), CD278/ICOS, IgG2, IgG3, CD1c, CD4, CD8, CD69 and CCR4 were purchased from Biolegend. Antibodies against IgA1 and IgA2 were purchased from SouthernBiotech. All were conjugated with Maxpar® X8 Antibody Labeling Kit except IgA2, CD141, CD69 and CCR6 who were labelled using Maxpar MCP9 Antibody Labeling Kit. All other antibodies were purchased from Fluidigm/DVS. DNA positive cells were assessed using Cell-ID Intercalator-Ir (#201192B) from fluidigm. Panel 2: 113In-conjugated anti-CD8 (RPA-T8), 115In-conjugated anti-CD4 (RPA-T4), 149Sm-conjugated anti-CCR4 (L291H4), 176Yb-conjugated anti-CD127 (A019D5), 141Pr-conjugated anti-CCR6 (G034E3), 154Sm-conjugated anti-CXCR3 (G025H7), 168Er-conjugated anti-CCR9 (L053E8), 159Tb-conjugated anti-CCR7 (G043H7), 167Er-conjugated anti-CXCR5 (J252D4), 144Nd-conjugated anti-CCR5 (NP-6G4), 106Cd-conjugated anti-CD45 (HI30), 111Cd-conjugated

anti-CD3 (UCHT1), 142Nd-conjugated anti-CD44 (IM7), 158Gd-conjugated anti-CD25 (M-A251), 141Pr-conjugated anti-CCR6 (G034E3), 163Dy-conjugated anti-CD38 (HIT2), 153Eu-conjugated anti-TIGIT (MBSA43), 147Sm-conjugated anti-2B4 (C1.7), 151Eu-conjugated anti-PD1 (EH12.2H7), 155Gd-conjugated anti-CD27 (L128), 162Dy-conjugated anti-CD69 (FN50), 164Dy-conjugated anti-CD45RO (UCHL1), 209Bi-conjugated anti-CD16 (3G8), 145Nd-conjugated anti-CD31 (WM59), 161Dy-conjugated anti-CD95 (DX2), 194Pt-conjugated anti-CD57 (NK-1), 166Er-conjugated anti-NKG2D (ON72), 170Er-conjugated anti-CD45RA (HI100), 174Yb-conjugated anti-HLADR (L243), 148Nd-conjugated anti-PDL1 (29E.2A3), 171Yb-conjugated anti-CD151 (50/6), 152Sm-conjugated anti-CD40L (TRAP1), 143Nd-conjugated anti-ICOS (C398.4A), 172Yb-conjugated anti-LAG3 (874501), 150Nd-conjugated anti-OX40 (ACT35), 160Gd-conjugated anti-Tbet (4B10), 165Ho-conjugated anti-Ki67 (Ki67), 169Tm-conjugated anti-Bcl2 (100), 175Lu-conjugated anti-RorγT (AFKJS-9), 146Nd-conjugated anti-Gata3 (TWAJ), 156Gd-conjugated anti-FoxP3 (PCH101) and 191Ir was used to label DNA. Antibodies against CD45, CD8, CD4, CD44, ICOS, 2B4, PD-1, CXCR3, CD25, CCR7, CD38, Ki67, CXCR5, Bcl2 were purchased from Biolegend. Antibodies against CD3, CD40L (CD154), CD57 and CD151 were purchased from BD. Antibodies against Gata3, FoxP3, CD95(FAS) and RorγT were purchased from e-Biosciences. Antibody against LAG3 was purchased from R&D Systems. All were conjugated with Maxpar® X8 Antibody Labeling Kit except CD45 and CD3 who were labelled using Maxpar MCP9 Antibody Labeling Kit. All other antibodies were purchased from Fluidigm/DVS. DNA positive cells were assessed using Cell-ID Intercalator-Ir (#201192B) from fluidigm. Panel 3: 154Sm -conjugated anti-CD3 (UCHT1), 106Cd -conjugated anti-CD45 (HI30), 113In -conjugated anti-CD8 (RPA-T8), 115In -conjugated anti-CD4 (RPA-T4), 142Nd -conjugated anti-CD19 (HIB19), 143Nd -conjugated anti-CD1c (L161) , 144Nd -conjugated anti-CD69 (FN50), 145Nd -conjugated anti-CD31 (WM59), 146Nd -conjugated anti-CD86 (GL-1) , 147Sm -conjugated anti-CD7 (CD7-6B7), 148Nd -conjugated anti-CD39 (A1) , 149Sm -conjugated anti-CD56 (HCD56), 150Nd -conjugated anti-pSTAT5 (47), 151Eu-conjugated anti-CD123 (6H6), 152Sm -conjugated anti-CD21 (BL13), 153Eu -conjugated anti-pSTAT1 [Y701] (58D6), 155Gd -conjugated anti-CD27 (L128), 156Gd -conjugated anti-p38 [T180/Y182] (D3F9), 158Gd -conjugated anti-pSTAT3 (4/P-Stat3), 159Tb -conjugated anti-pMAPKAPK2 (27B7), 160Gd -conjugated anti-CD14 (M5E2), 162Dy -conjugated anti-CD11c (Bu15), 163Dy -conjugated anti-CD62L (DREG-56) , 164Dy -conjugated anti-CD161 (HP3G10), 165Ho -conjugated anti-pNFkb (K10895.12.50) , 166Er -conjugated anti-CD20 (2H7), 167Er -conjugated anti-CD38 (HIT2), 168Er -conjugated anti-Ki67 (Ki67) , 169Tm -

conjugated anti-CD45RA (HI100) , 171Yb -conjugated anti-pERK1/2 [T202/Y204] (D13.14.4E), 172Yb -conjugated anti-CD15 [SSEA-1] (W6D3), 173Yb -conjugated anti-CD141 (1A4), 174Yb -conjugated anti-HLA-DR (L243), 175Lu -conjugated anti-pS6 (N7548), 176Yb -conjugated anti-pCREB (87G3), 194Pt -conjugated anti-CD57 (NK-1), 209Bi -conjugated anti-CD16 (3G8) and 191Ir was used to label DNA. Antibodies against Ki67, CD45, CD8a, CD4, CD1c, CD69, CD86, CD39, CD56, CD62L and CD45RA were purchased from Biolegend. Antibodies against NF-kB p65, CD20, CD141 and CD57 were purchased from BD. All were conjugated with Maxpar® X8 Antibody Labeling Kit except CD45 which was labelled using Maxpar MCP9 Antibody Labeling Kit. All other antibodies were purchased from Fluidigm/DVS. DNA positive cells were assessed using Cell-ID Intercalator-Ir (#201192B) from fluidigm. Additional information regarding Abs provider, reference number and titration are available in Supplementary Data 1.

Supplementary Figure 1

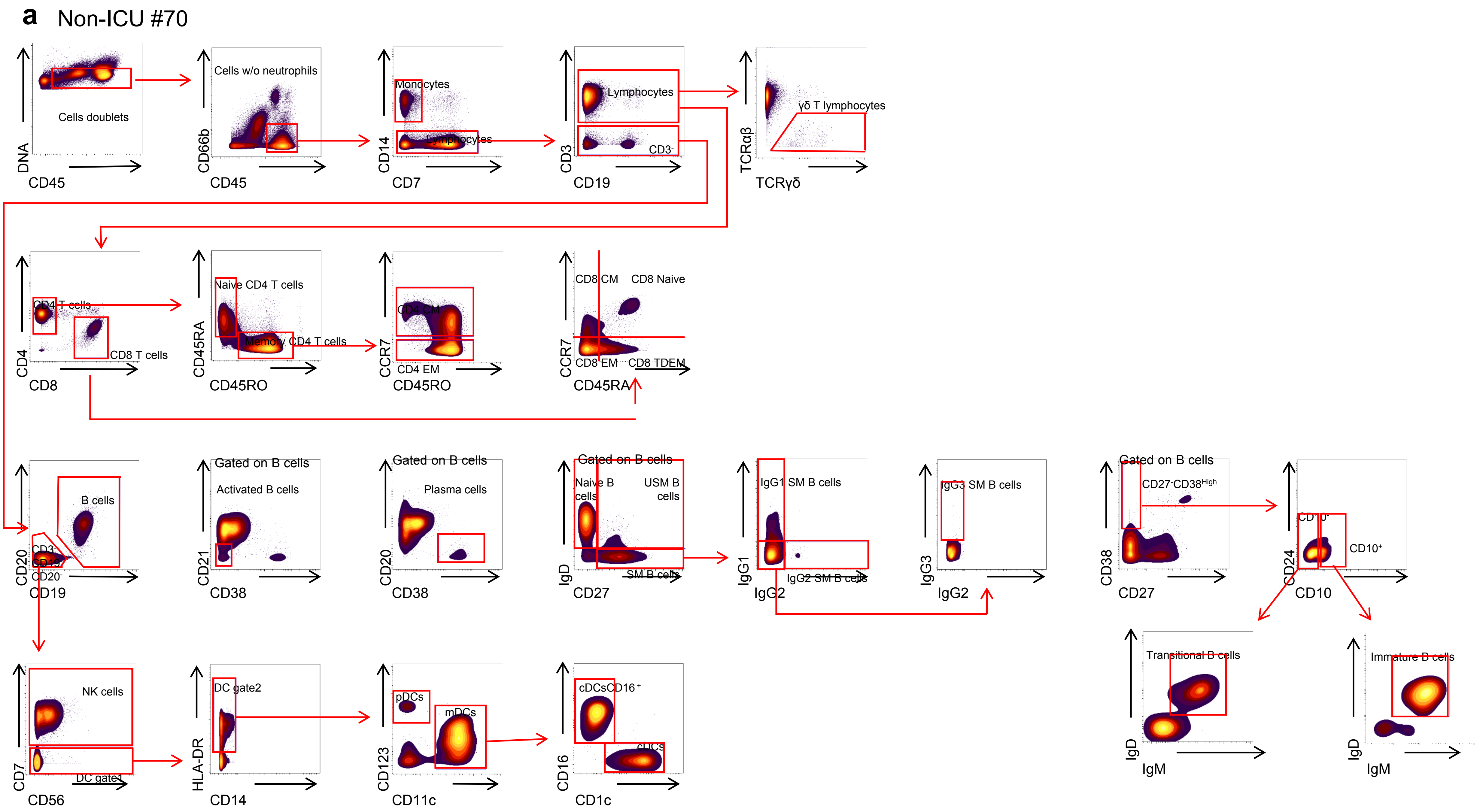

b Non-ICU #70

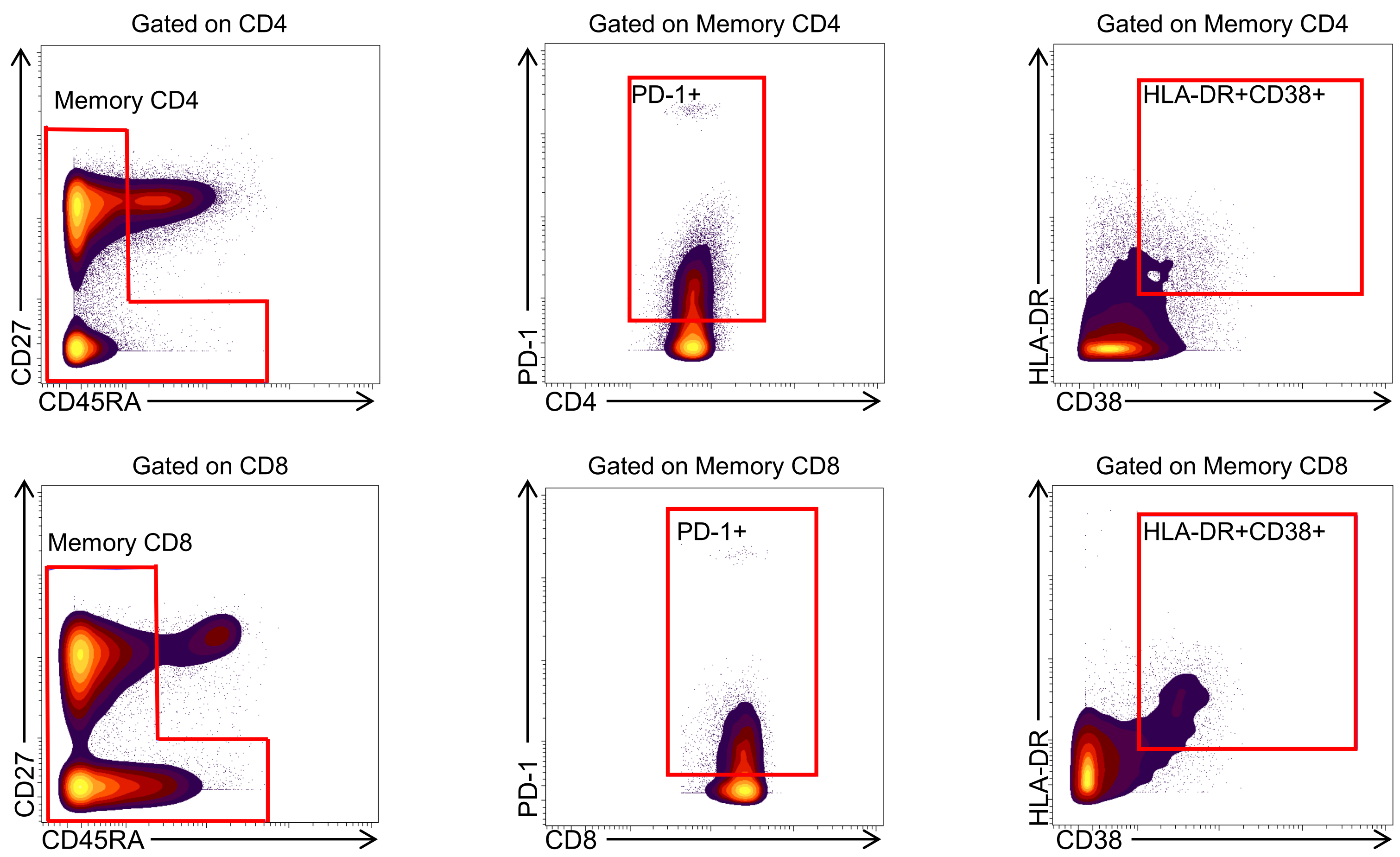
Gated on CD4
Memory CD4
CD27
CD45RA
Gated on Memory CD4
PD-1+
PD-1
CD4
Gated on Memory CD4
HLA-DR+CD38+
HLA-DR
CD38
Gated on CD8
Memory CD8
CD27
CD45RA
Gated on Memory CD8
PD-1+
PD-1
CD8
Gated on Memory CD8
HLA-DR+CD38+
HLA-DR
CD38

c

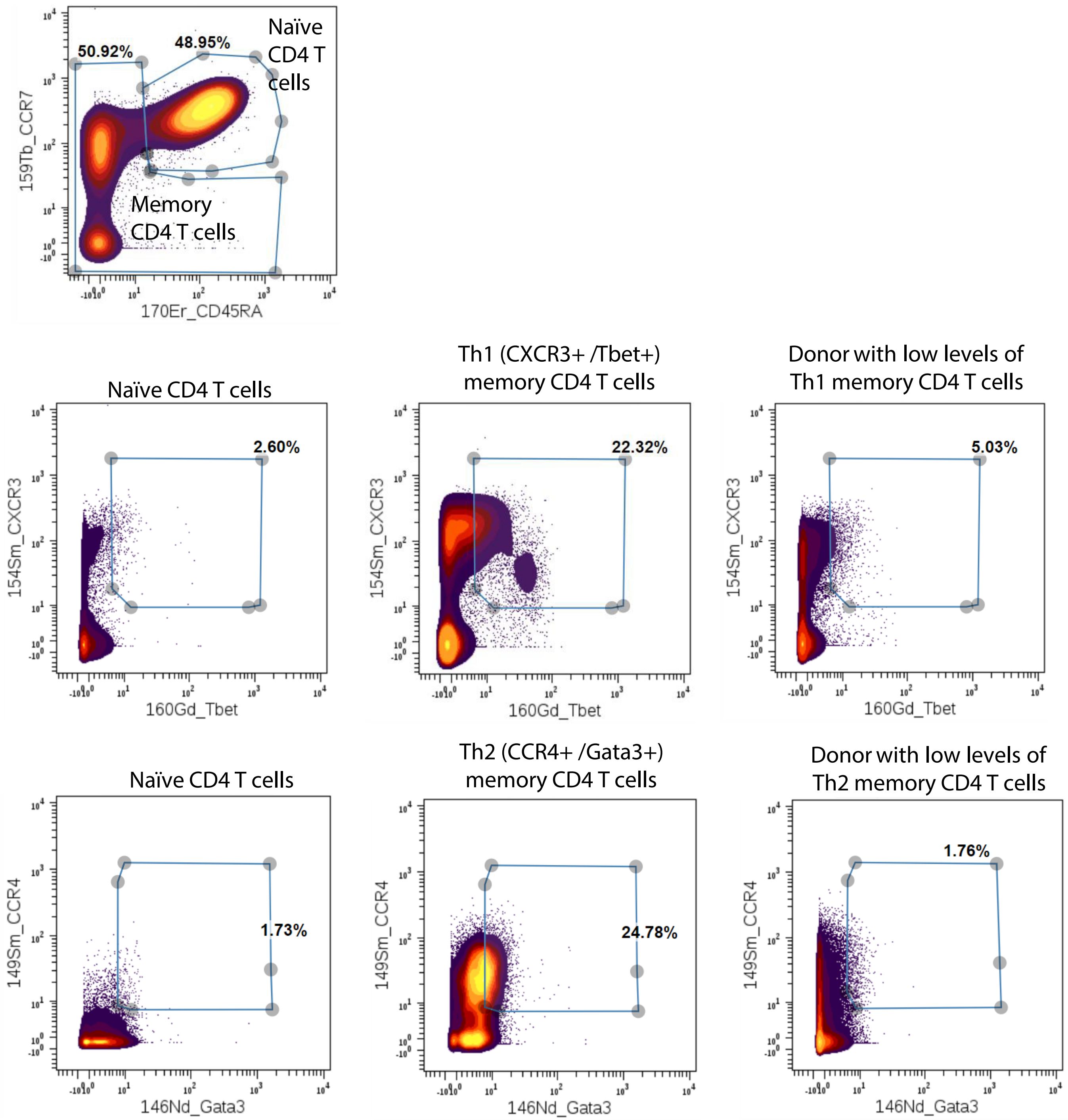
Naïve CD4 T cells
50.92%
48.95%
Memory CD4 T cells
159Tb_CCR7
170Er_CD45RA
Naïve CD4 T cells
2.60%
Th1 (CXCR3+ /Tbet+) memory CD4 T cells
22.32%
Donor with low levels of Th1 memory CD4 T cells
5.03%
154Sm_CXCR3
160Gd_Tbet
Naïve CD4 T cells
1.73%
Th2 (CCR4+ /Gata3+) memory CD4 T cells
24.78%
Donor with low levels of Th2 memory CD4 T cells
1.76%
149Sm_CCR4
146Nd_Gata3

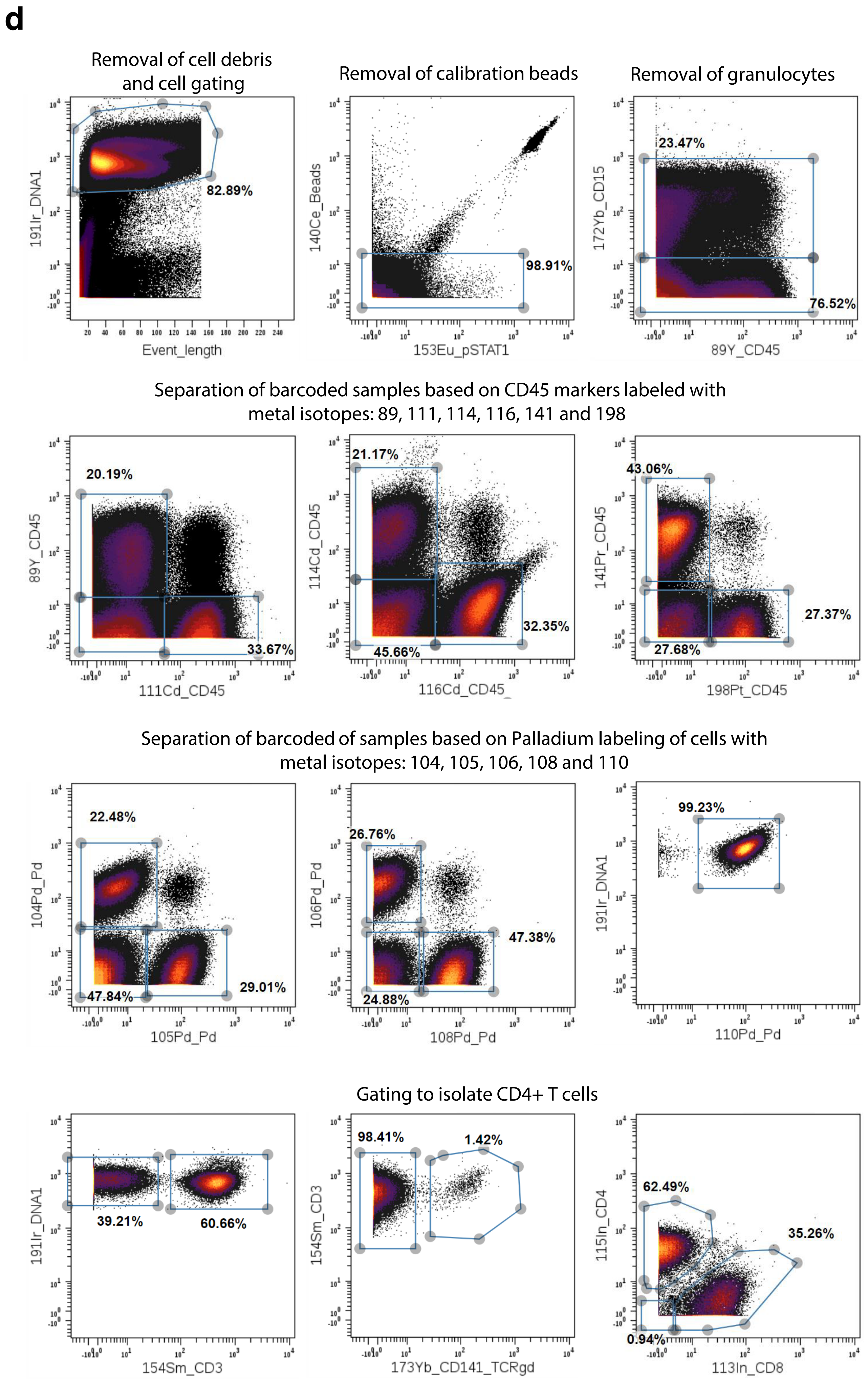

Supplementary Figure 1. Gating strategies.

a. Gating strategy used for Supplementary Figure 2, **b**. Gating strategy used for Supplemental Figure 3, **c**. Gating strategy used for Figure 1a and **d**. Gating strategy used for Figure 1b.

Supplementary Figure 2

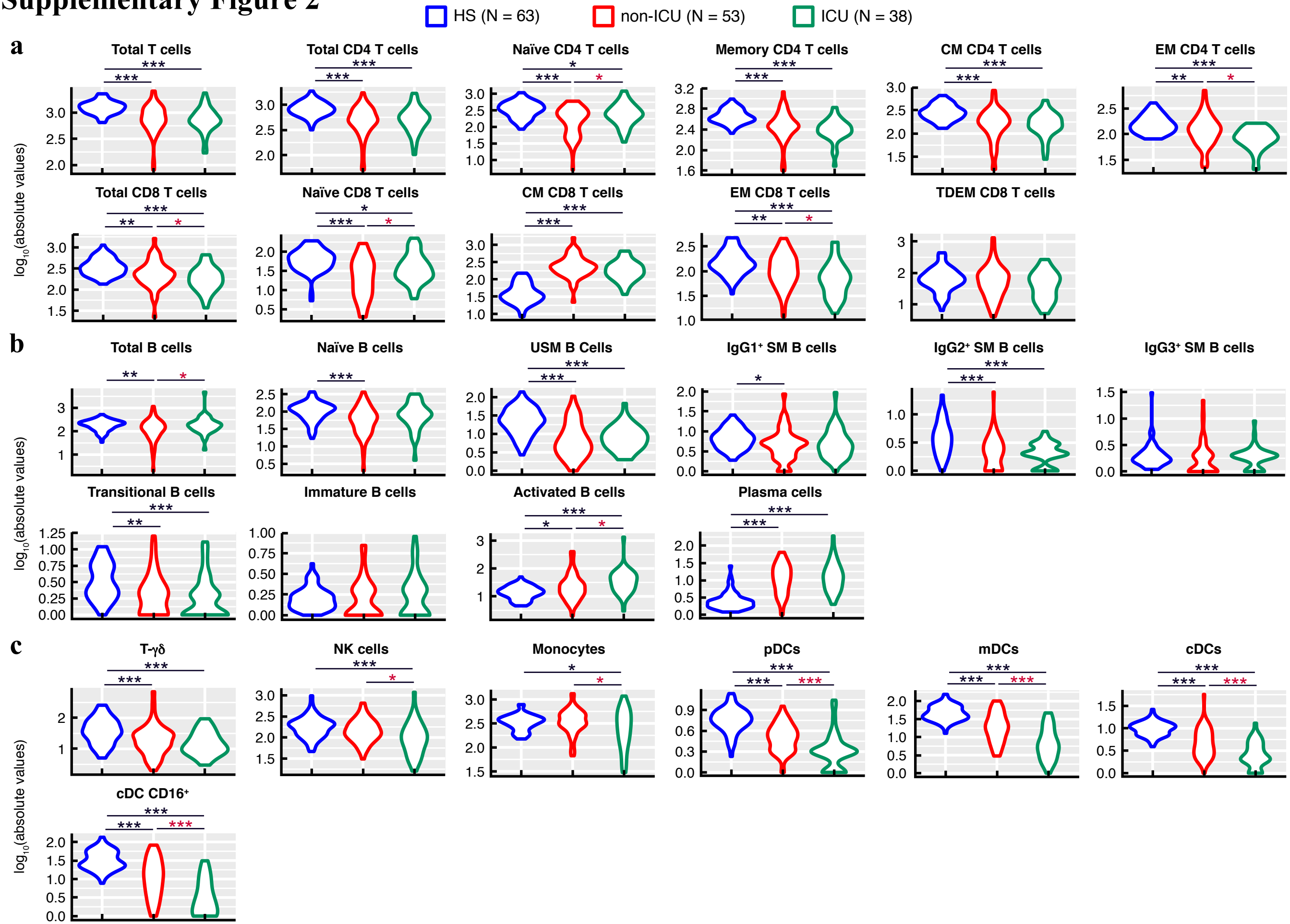

Supplementary Figure 2. Immune profile of circulating cell populations in ICU and non-ICU COVID-19 patients. a Absolute values of CD4 and CD8 T-cell sub-populations; **b** Absolute values of B-cell subpopulations; **c** Absolute values of gamma-delta T, NK, monocytic and dendritic cell populations in healthy subjects (N=63), non-ICU (N=53) and ICU (N=38) patients. Blue plots correspond to healthy subjects (H.S), red plots corresponds to non-ICU patients and green plots correspond to ICU patients (a-c). Black stars indicate statistical significance between ICU or non-ICU patients and healthy subjects. Red stars indicate statistical significance between ICU and non-ICU patients. Statistical significance (*P* values) was obtained using two-sided Kruskal-Wallis test, using a Bonferroni correction.*=P<0.05; **=P<0.01; ***=P<0.001. 'CM' corresponds to central memory, 'EM' corresponds to effector memory, 'TDEM' corresponds to terminally differentiated effector memory, 'USM' corresponds to unswitched memory, 'SM' corresponds to switched memory, 'pDC' corresponds to plasmacytoid dendritic cells, 'mDCs' corresponds to myeloid dendritic cells and 'cDCs' corresponds to conventional dendritic cells. Exact *P* values are available in Source Data file.

Supplementary Figure 3

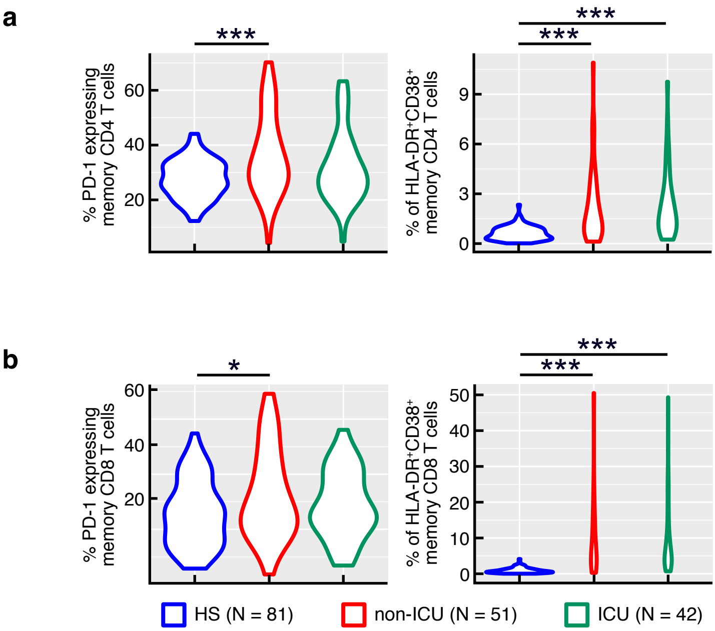

Supplementary Figure 3. Percentage of activated CD4 and CD8 T cells of non-ICU and ICU SARS-CoV-2 infected individuals. a. Ex vivo percentage of PD-1 or HLA-DR+CD38+ memory (CD45RA-CD45RO+) CD4 T cells in healthy subjects (N=81), non-ICU (N=51) and ICU (N=42) individuals. **b** Ex vivo percentage of PD-1 or HLA-DR+CD38+ memory (CD45RA-CD27-) CD8 T cells in healthy subjects (N=81), non-ICU (N=51) and ICU (N=42) individuals. Blue plots correspond to healthy subjects (H.S), red plots corresponds to non-ICU patients and green plots correspond to ICU patients. Red stars indicate statistical significance between ICU or non-ICU SARS-CoV2-infected individuals and healthy subjects. Statistical significance (*P* values) was obtained using two-sided Kruskal-Wallis test, using a Bonferroni correction.*=P<0.05; ***=P<0.001. Exact *P* values are available in Source Data file.

Supplementary Figure 4

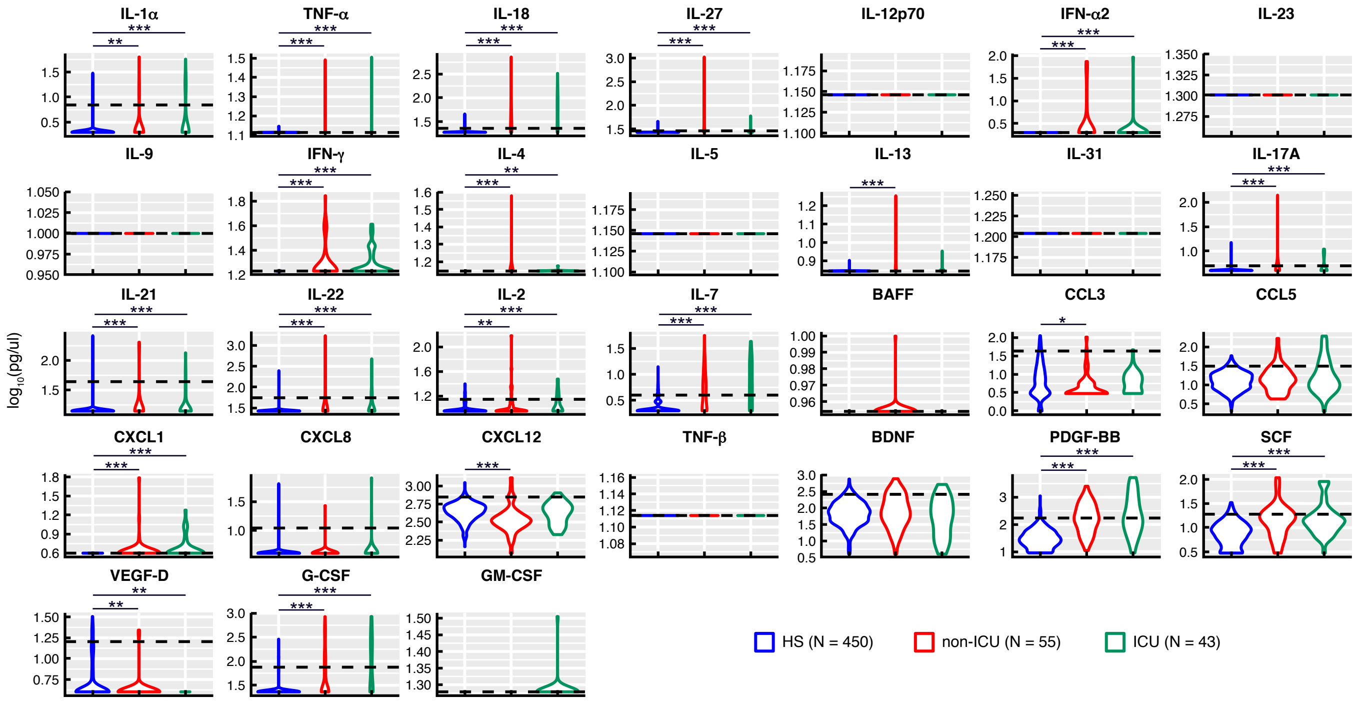

Supplementary Figure 4. Serum cytokine, soluble cytokine receptor, chemokine and growth factor profiles in non-ICU and ICU COVID-19 patients. Levels of cytokines (IL-1α, TNF-α, IL-18, IL-27, IL-12p70, IFN-α2, IL-23, IL-9, IFN-γ, IL-4, IL-5, IL-13, IL-31, IL-17A, IL-21, IL-22, IL-2, IL-7, BAFF, TNF-β), chemokines (CCL3, CCL5, CXCL1, CXCL8, CXCL12) and growth factors (BDNF, PDGF-BB, SCF, VEGF-D, G-CSF, GM-CSF) in healthy subjects (N=450), non-ICU (N=55) and ICU (N=43) patients. Blue plots correspond to healthy subjects (H.S), red plots corresponds to non-ICU patients and green plots correspond to ICU patients. Dotted line represents the upper normal values. Black stars indicate statistical significance between ICU or non-ICU patients and healthy subjects. Statistical significance (P values) was obtained using two-sided Kruskal-Wallis test, using a Bonferroni correction. *=P<0.05; **=P<0.01; ***=P<0.001. Exact *P* values are available in Source Data file.

Supplemental Figure 5

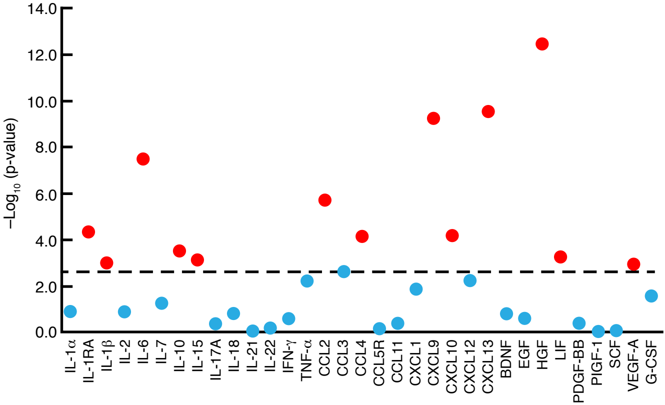

Supplementary Figure 5. Serum factors differently distributed between ICU and non-ICU individuals enrolled in the investigational cohort. Statistical significance (*P* values) was obtained using two-sided Kruskal-Wallis test. Bonferroni's correction was applied for multiple comparison. Red circles correspond to serum markers with P-value below the threshold ($P<0.05$). Blue circles correspond to serum markers with P-value above the threshold. Exact *P* values are available in Source Data file.

French COVID cohort study group

Laurent ABEL (Inserm UMR 1163, Paris, France), Claire ANDREJAK (CHU Amiens, France), François ANGOULVANT (Hôpital Necker, Paris, France), Delphine BACHELET, Krishna BHAVSAR, Lila BOUADMA, Anissa CHAIR, Camille COUFFIGNAL, Charlene DA SILVEIRA, Marie-Pierre DEBRAY, Diane DESCAMPS, Xavier DUVAL, Philippine ELOY, Marina ESPOSITO-FARESE, Nadia ETTALHAOUI, Nathalie GAULT, Jade GHOSN, Isabelle GORENNE, Isabelle HOFFMANN, Ouifiya KAFIF, Sabrina KALI, Antoine KHALIL, Cédric LAOUÉNAN, Samira LARIBI, Minh LE, Quentin LE HINGRAT, François-Xavier LESCURE, Jean Christophe LUCET, France MENTRÉ, Jimmy Mullaert, Nathan PEIFFER-SMADJA, Gilles PEYTAVIN, Carine ROY, Marion SCHNEIDER, Nassima SI MOHAMMED, Lysa TAGHERSET, Coralie TARDIVON, Marie-Capucine TELLIER, Jean-François TIMSIT, Théo TRIOUX, Sarah TUBIANA, Benoit VISSEAUX, Yazdan YAZDANPANAH (Hôpital Bichat, Paris, France), Romain BASMACI, Olivier PICONE (Hôpital Louis Mourier, Colombes, France), Sylvie BEHILILL, Sylvie VAN DER WERF, Vincent ENOUF, Hugo MOUQUET (Pasteur Institute, Paris, France), Marine BELUZE (F-CRIN Partners Platform, Paris, France), Dehbia BENKERROU, Céline DORIVAL, François TÉOULÉ, Amina MEZIANE (Inserm UMR 1136, Paris, France), François BOMPART (Drugs for Neglected Diseases initiative, Geneva, Switzerland), Maude BOUSCAMBERT (Inserm UMR 1111, Lyon, France), Minerva CERVANTES-GONZALEZ, Eric d'ORTENZIO, Oriane PUÉCHAL, Caroline SEMAILLE (REACTing, Paris, France), Catherine CHIROUZE (CHRU Jean Minjoz, Besançon, France), Alexandra COELHO (Inserm UMR 1018, Paris, France), Sandrine COUFFIN-CADIERGUES, Hélène ESPEROU, Ikram HOUAS, Salma JAAFOURA, Aurélie PAPADOPOULOS (Inserm sponsor, Paris, France), Dominique DEPLANQUE (Hôpital Calmette, Lille, France), Mathilde DESVALLÉE, Coralie KHAN (Inserm UMR 1219, Bordeaux, France), Alpha DIALLO, Marie BARTOLI, Soizic LE MESTRE, Noémie MERCIER, Christelle PAUL, Ventzislava PETROV-SANCHEZ (ANRS, Paris, France), Alphonsine DIOUF, Alexandre HOCTIN, Marina MAMBERT (Inserm UMR 1018, Paris, France), François DUBOS (CHU Lille, France), Manuel ETIENNE (CHU Rouen, France), Alexandre GAYMARD (Inserm UMR 1111, Lyon, France), Tristan GIGANTE, Morgane GILG, Bénédicte ROSSIGNOL (F-CRIN INI-CRCT, Nancy, France), Jérémie GUEDJ, Hervé LE NAGARD, Guillaume LINGAS, Nadège NEANT (Inserm UMR 1137, Paris, France), Jean-Sébastien HULOT (Hôpital Européen Georges Pompidou, Paris, France), Florentia KAGUELIDOU, Justine PAGES (Hôpital Robert Debré, Paris, France), Yves LEVY, Aurélie WIEDEMANN (Vaccine Research Insitute (VRI), Inserm UMR 955, Créteil, France),

Claire LEVY-MARCHAL (F-CRIN INI-CRCT, Paris, France), Bruno LINA, Manuel ROSA-CALATRAVA, Olivier TERRIER (Inserm UMR 1111, Lyon, France), Denis MALVY (CHU Bordeaux, France), Marion NORET (RENARCI, Annecy, France), Patrick ROSSIGNOL (CHU Nancy, France), Christelle TUAL, Aurélie VEISLINGER (Inserm CIC-1414, Rennes, France), Noémie VANEL (Hôpital la Timone, Marseille, France)
