# Supplementary material for: The cytokines HGF and CXCL13 predict the severity and the mortality in COVID-19 patients
Source: Nat Commun. 2021 Aug 9;12:4888. doi: 10.1038/s41467-021-25191-5 (PMC8352963; doi:10.1038/s41467-021-25191-5)
Supplement: Supplementary file 3 — Description of Additional Supplementary Files [file 41467_2021_25191_MOESM3_ESM.pdf]

Description of Additional Supplementary Files

Supplementary Data 1: Contains detailed antibodies information of Panels 1-3 used in this study: isotope, marker, clone, provider, reference and dilution if relevant. Panel 1 was used to characterize circulating cell populations, Panel 2 for CD4 T cell lineage distribution and Panel 3 for CD4 T cell phospho-protein signaling profile.
