# Supplementary material for: The cytokines HGF and CXCL13 predict the severity and the mortality in COVID-19 patients
Source: Nat Commun. 2021 Aug 9;12:4888. doi: 10.1038/s41467-021-25191-5 (PMC8352963; doi:10.1038/s41467-021-25191-5)
Supplement: Supplementary file 5 — Reporting summary [file 41467_2021_25191_MOESM5_ESM.pdf]

nature research

Corresponding author(s): Giuseppe Pantaleo

Last updated by author(s): 27.05.2021

Reporting Summary

Nature Research wishes to improve the reproducibility of the work that we publish. This form provides structure for consistency and transparency in reporting. For further information on Nature Research policies, see our Editorial Policies and the Editorial Policy Checklist.

Statistics

For all statistical analyses, confirm that the following items are present in the figure legend, table legend, main text, or Methods section.

n/a	Confirmed	
☐	☒	The exact sample size (n) for each experimental group/condition, given as a discrete number and unit of measurement
☐	☒	A statement on whether measurements were taken from distinct samples or whether the same sample was measured repeatedly
☐	☒	The statistical test(s) used AND whether they are one- or two-sided<br>*Only common tests should be described solely by name; describe more complex techniques in the Methods section.*
☐	☒	A description of all covariates tested
☐	☒	A description of any assumptions or corrections, such as tests of normality and adjustment for multiple comparisons
☐	☒	A full description of the statistical parameters including central tendency (e.g. means) or other basic estimates (e.g. regression coefficient) AND variation (e.g. standard deviation) or associated estimates of uncertainty (e.g. confidence intervals)
☐	☒	For null hypothesis testing, the test statistic (e.g. F, t, r) with confidence intervals, effect sizes, degrees of freedom and P value noted<br>*Give P values as exact values whenever suitable.*
☒	☐	For Bayesian analysis, information on the choice of priors and Markov chain Monte Carlo settings
☒	☐	For hierarchical and complex designs, identification of the appropriate level for tests and full reporting of outcomes
☒	☐	Estimates of effect sizes (e.g. Cohen's d, Pearson's r), indicating how they were calculated

Our web collection on statistics for biologists contains articles on many of the points above.

Software and code

Policy information about availability of computer code

Data collection	flowjo version 10.7.1 and Fluidigm Cytobank software package version 7.3.0 were used to analyse the data.
Data analysis	Stata version 16.1 for Windows (Stata Corp, College Station, TX, USA) or R software version 3.6.3. No custom code has been developed and used in the study. The codes used in the present study are available in R Stats package version 3.6.3.

For manuscripts utilizing custom algorithms or software that are central to the research but not yet described in published literature, software must be made available to editors and reviewers. We strongly encourage code deposition in a community repository (e.g. GitHub). See the Nature Research guidelines for submitting code & software for further information.

Data

Policy information about availability of data

All manuscripts must include a data availability statement. This statement should provide the following information, where applicable:
- Accession codes, unique identifiers, or web links for publicly available datasets
- A list of figures that have associated raw data
- A description of any restrictions on data availability

The authors declare that all other data supporting the findings of this study are available within the paper and its supplementary information files.

Field-specific reporting

Life sciences study design

All studies must disclose on these points even when the disclosure is negative.

Sample size	The sample size was estimated based on a previously published study (Laing, A.G., et al. A dynamic COVID-19 immune signature includes associations with poor prognosis. Nat Med 26, 1623-1635 (2020))
Data exclusions	No data were excluded
Replication	We used one test cohort and two independent validation cohorts. All attempts at replication were successful
Randomization	The samples were not ramdomized. Allocation to ICU and non-ICU groups was based on the inclusion criteria described in the Method section.
Blinding	The data were collected from 3 cohorts i.e. the LUH discovery cohort and 2 validation cohorts LUH-2 and FCS. The physicians were not blinded during the enrolement, while the investigators were blinded during the data collection but not at the time of data analysis. Regarding the validation cohorts, the investigators were blinded during both the data collection and data analysis. The aforementioned strategy is commonly used to identify potential biomarkers differentiating groups of patients.

Reporting for specific materials, systems and methods

We require information from authors about some types of materials, experimental systems and methods used in many studies. Here, indicate whether each material, system or method listed is relevant to your study. If you are not sure if a list item applies to your research, read the appropriate section before selecting a response.

Materials & experimental systems

n/a	Involved in the study	
☐	☒	Antibodies
☒	☐	Eukaryotic cell lines
☒	☐	Palaeontology and archaeology
☒	☐	Animals and other organisms
☐	☒	Human research participants
☒	☐	Clinical data
☒	☐	Dual use research of concern

Methods

n/a	Involved in the study	
☒	☐	ChIP-seq
☒	☐	Flow cytometry
☒	☐	MRI-based neuroimaging

Antibodies

Antibodies used

Information (supplier name, catalog number and clone name) of all antibodies used in the present study are available in the Supplemental Material.

The following antibodies were used for mass cytometry experiments. Panel 1: 111Cd-conjugated anti-CD141 (1A4), 113In-conjugated anti-CD8 (RPA-T8), 115In-conjugated anti-CD4 (RPA-T4), 116Cd-conjugated anti-IgA2 (A9604D2), 141Pr-conjugated anti-CD45 (HI30), 142Nd-conjugated anti-CD19 (HIB19), 143Nd-conjugated anti-ICOS (C398.4A), 144Nd-conjugated anti-IgG3 (HP6047), 145Nd-conjugated anti-CD31/PECAM-1 (WM59), 146Nd-conjugated anti-IgD (IA6-2), 147Sm-conjugated anti-CD7 (CD7-6B7), 148Nd-conjugated anti-IgA1 (B3506B4), 149Sm-conjugated anti-CD127 (A019D5), 150Nd-conjugated anti-IgG1 (G17-1), 151Eu-conjugated anti-CD123 (6H6), 152Sm-conjugated anti-CD21 (BL13), 153Eu-conjugated anti-CD62L (DREG-56), 154Sm-conjugated anti-CD3 (UCHT1), 155Gd-conjugated anti-CD27 (L128), 156Gd-conjugated anti-TCR γ (gamma)/δ (delta) (B1), 158Gd-conjugated anti-CD10 (HI10a), 159Tb-conjugated anti-CD197/CCR7 (G043H7), 160Gd-conjugated anti-CD14 (M5E2), 161Dy-conjugated anti-CD1c (L161), 162Dy-conjugated anti-CD11c (Bu15), 163Dy-conjugated anti-CD183/CXCR3 (G025H7), 164Dy-conjugated anti-CD185/CXCR5 (51505), 165Ho-conjugated anti-CD45RO (UCHL1), 166Er-conjugated anti-CD24 (ML5), 167Er -conjugated anti-CD38 (HIT2), 168Er-conjugated anti-CD66b (G10F5), 169Tm-conjugated anti-CD25 (2A3), 170Er-conjugated anti-CD45RA (HI100), 171Yb-conjugated anti-CD20 (2H7), 172Yb-conjugated anti-IgM (MHM-88), 173Yb-conjugated anti-TCR α(alpha)/ β(beta) (T1089.A-31), 174Yb-conjugated anti-HLA-DR (L243), 175Lu-conjugated anti-CD279/PD-1 (EH12.2H7), 176Yb-conjugated anti-CD56 (HCD56), 198Pt-conjugated anti-IgG2 (HP6002), 209Bi-conjugated anti-CD16 (3G8), 112Cd-conjugated anti-CD69 (FN50), 106Cd-conjugated anti-CCR6 (11A9), 194Pt-conjugated anti-CCR4 (L291H4) and 191Ir was used to label DNA. Antibodies against TCR α(alpha)/ β(beta), IgG1, CD66b, CCR6 and CD141 were purchased from BD. Antibodies against TCR γ (gamma)/δ (delta), CD278/ICOS, IgG2, IgG3, CD1c, CD4, CD8, CD69 and CCR4 were purchased from Biolegend. Antibodies against IgA1 and IgA2 were purchased from SouthernBiotech. All were conjugated with Maxpar® X8 Antibody Labeling Kit except IgA2, CD141, CD69 and CCR6 who were labelled using Maxpar MCP9 Antibody Labeling Kit. All other antibodies were purchased from Fluidigm/DVS. DNA positive cells were assessed using

Cell-ID Intercalator-Ir (#201192B) from fluidigm. Panel 2: 113In-conjugated anti-CD8 (RPA-T8), 115In-conjugated anti-CD4 (RPA-T4), 149Sm-conjugated anti-CCR4 (L291H4), 176Yb-conjugated anti-CD127 (A019D5), 141Pr-conjugated anti-CCR6 (G034E3), 154Sm-conjugated anti-CXCR3 (G025H7), 168Er-conjugated anti-CCR9 (L053E8), 159Tb-conjugated anti-CCR7 (G043H7), 167Er-conjugated anti-CXCR5 (J252D4), 144Nd-conjugated anti-CCR5 (NP-6G4), 106Cd-conjugated anti-CD45 (HI30), 111Cd-conjugated anti-CD3 (UCHT1), 142Nd-conjugated anti-CD44 (IM7), 158Gd-conjugated anti-CD25 (M-A251), 141Pr-conjugated anti-CCR6 (G034E3), 163Dy-conjugated anti-CD38 (HIT2), 153Eu-conjugated anti-TIGIT (MBSA43), 147Sm-conjugated anti-2B4 (C1.7), 151Eu-conjugated anti-PD1 (EH12.2H7), 155Gd-conjugated anti-CD27 (L128), 162Dy-conjugated anti-CD69 (FN50), 164Dy-conjugated anti-CD45RO (UCHL1), 209Bi-conjugated anti-CD16 (3G8), 145Nd-conjugated anti-CD31 (WM59), 161Dy-conjugated anti-CD95 (DX2), 194Pt-conjugated anti-CD57 (NK-1), 166Er-conjugated anti-NKG2D (ON72), 170Er-conjugated anti-CD45RA (HI100), 174Yb-conjugated anti-HLADR

(L243), 148Nd-conjugated anti-PDL1 (29E.2A3), 171Yb-conjugated anti-CD151 (50/6), 152Sm-conjugated anti-CD40L (TRAP1), 143Nd-conjugated anti-ICOS (C398.4A), 172Yb-conjugated anti-LAG3 (874501), 150Nd-conjugated anti-OX40 (ACT35), 160Gd-conjugated anti-Tbet (4B10), 165Ho-conjugated anti-Ki67 (Ki67), 169Tm-conjugated anti-Bcl2 (100), 175Lu-conjugated anti-RorγT (AFKJS-9), 146Nd-conjugated anti-Gata3 (TWAJ), 156Gd-conjugated anti-FoxP3 (PCH101) and 191Ir was used to label DNA. Antibodies against CD45, CD8, CD4, CD44, ICOS, 2B4, PD-1, CXCR3, CD25, CCR7, CD38, Ki67, CXCR5, Bcl2 were purchased from Biolegend. Antibodies against CD3, CD40L (CD154), CD57 and CD151 were purchased from BD. Antibodies against Gata3, FoxP3, CD95(FAS) and RorγT were purchased from e-Biosciences. Antibody against LAG3 was purchased from R&D Systems. All were conjugated with Maxpar® X8 Antibody Labeling Kit except CD45 and CD3 who were labelled using Maxpar MCP9 Antibody Labeling Kit. All other antibodies were purchased from Fluidigm/DVS. DNA positive cells were assessed using Cell-ID Intercalator-Ir (#201192B) from fluidigm. Panel 3: 154Sm -conjugated anti-CD3 (UCHT1), 106Cd -conjugated anti-CD45 (HI30), 113In -conjugated anti-CD8 (RPA-T8), 115In -conjugated anti-CD4 (RPA-T4), 142Nd -conjugated anti-CD19 (HIB19), 143Nd -conjugated anti-CD1c (L161) , 144Nd -conjugated anti-CD69 (FN50), 145Nd -conjugated anti-CD31 (WM59), 146Nd -conjugated anti-CD86 (GL-1) , 147Sm -conjugated anti-CD7 (CD7-6B7), 148Nd -conjugated anti-CD39 (A1) , 149Sm -conjugated anti-CD56 (HCD56), 150Nd -conjugated anti-pSTAT5 (47), 151Eu-conjugated anti-CD123 (6H6), 152Sm -conjugated anti-CD21 (BL13), 153Eu -conjugated anti-pSTAT1 [Y701] (58D6), 155Gd -conjugated anti-CD27 (L128), 156Gd -conjugated anti-p38 [T180/Y182] (D3F9), 158Gd -conjugated anti-pSTAT3 (4/P-Stat3), 159Tb -conjugated anti-pMAPKAPK2 (27B7), 160Gd -conjugated anti-CD14 (M5E2), 162Dy -conjugated anti-CD11c (Bu15), 163Dy -conjugated anti-CD62L (DREG-56) , 164Dy -conjugated anti-CD161 (HP3G10), 165Ho -conjugated anti-pNFkb (K10895.12.50) , 166Er -conjugated anti-CD20 (2H7), 167Er -conjugated anti-CD38 (HIT2), 168Er -conjugated anti-Ki67 (Ki67) , 169Tm -conjugated anti-CD45RA (HI100) , 171Yb -conjugated anti-pERK1/2 [T202/Y204] (D13.14.4E), 172Yb -conjugated anti-CD15 [SSEA-1] (W6D3), 173Yb -conjugated anti-CD141 (1A4), 174Yb -conjugated anti-HLA-DR (L243), 175Lu -conjugated anti-pS6 (N7548), 176Yb -conjugated anti-pCREB (87G3), 194Pt -conjugated anti-CD57 (NK-1), 209Bi -conjugated anti-CD16 (3G8) and 191Ir was used to label DNA. Antibodies against Ki67, CD45, CD8a, CD4, CD1c, CD69, CD86, CD39, CD56, CD62L and CD45RA were purchased from Biolegend. Antibodies against NF-kB p65, CD20, CD141 and CD57 were purchased from BD. All were conjugated with Maxpar® X8 Antibody Labeling Kit except CD45 which was labelled using Maxpar MCP9 Antibody Labeling Kit. All other antibodies were purchased from Fluidigm/DVS. DNA positive cells were assessed using Cell-ID Intercalator-Ir (#201192B) from fluidigm. Additional information regarding Abs provider, reference number and titration are available in Supplementary Dataset 3.

Validation

Accoding to the manufacturer's website (www.fluidigm.com), all antibodies were validated as follows: Each lot of conjugated antibody was quality control tested by CyTOF® analysis of stained cells using the appropriate positive and negative cell staining and/or activation controls.

Human research participants

Policy information about studies involving human research participants

Population characteristics

The population characteristics are depicted in Supplemental Table1.

Recruitment

Adult participants were recruited by clinicians in ICU and internal medicine ward on the basis on SARS-CoV-2 positive PCR. Only pregnant women were excluded. Due to the nature of the recruitement, we estimate that there were no self-selection bias or any other bias in the recruitement of the volunteers in the present study.

Ethics oversight

The "Commission cantonale (VD) d'éthique de la recherche sur l'être humain (CER-VD)" approved this study

Note that full information on the approval of the study protocol must also be provided in the manuscript.
